# Ultrasonographic evaluation of FMF patients with exertional leg pain: an overlooked component of the disease

**DOI:** 10.1093/rheumatology/keag226

**Published:** 2026-04-24

**Authors:** Batuhan Küçükali, Çisem Yıldız, Nuran Belder, Merve Kutlar Tanıdır, Büşra Acun, Khadija Ahmadova, Nihal Karaçayır, Deniz Gezgin Yıldırım, Sevcan A Bakkaloğlu

**Affiliations:** Department of Pediatric Rheumatology, Gazi University Faculty of Medicine, Ankara, Turkey; Department of Pediatric Rheumatology, Gazi University Faculty of Medicine, Ankara, Turkey; Department of Pediatric Rheumatology, Gazi University Faculty of Medicine, Ankara, Turkey; Department of Pediatric Rheumatology, Gazi University Faculty of Medicine, Ankara, Turkey; Department of Pediatric Rheumatology, Gazi University Faculty of Medicine, Ankara, Turkey; Department of Pediatric Rheumatology, Gazi University Faculty of Medicine, Ankara, Turkey; Department of Pediatric Rheumatology, Gazi University Faculty of Medicine, Ankara, Turkey; Department of Pediatric Rheumatology, Gazi University Faculty of Medicine, Ankara, Turkey; Department of Pediatric Rheumatology, Gazi University Faculty of Medicine, Ankara, Turkey

**Keywords:** exertional leg pain, FMF, musculoskeletal ultrasonography, exercise-induced myalgia

## Abstract

**Objectives:**

Exertional leg pain (ELP) is an underrecognized manifestation of FMF with unclear pathophysiology. This study performed a comprehensive ultrasonographic evaluation of lower-extremity joints, tendons and entheses in paediatric FMF patients with ELP, comparing findings with non-ELP patients and matched healthy controls.

**Methods:**

Paediatric FMF patients carrying homozygous or compound heterozygous pathogenic MEFV variants were enrolled in this prospective study. Demographic, anthropometric and clinical data were collected, while a blinded operator performed standardized ultrasonographic evaluations.

**Results:**

A total of 50 patients and 25 heathy controls were included (25 with ELP, 25 without). No differences were observed between the groups in demographic, anthropometric or FMF-related factors, including MEFV mutations and ISSF scores. Among the evaluated parameters, Achilles tendon thickness was significantly increased in children with ELP, independent of demographic or anthropometric factors, compared with both healthy and non-ELP group (*P* < 0.01). Nine patients exhibited grade 1 knee synovial effusions, predominantly in the ELP cohort, most with subclinical inflammation. One patient with ELP demonstrated enthesitis and was diagnosed with HLA-B27-negative ERA. Patellar tendon and plantar fascia thickness were comparable.

**Conclusion:**

Paediatric FMF patients with ELP demonstrated increased achilles tendon thickness. Although no standardized cut-off exists for tendinopathy, this may reflect tendon structural changes related to ELP. Moreover, FMF patients, particularly those with ELP, exhibited inter-attack knee effusions which may serve as a marker for subclinical inflammation. Larger longitudinal cohorts are needed to define ELP, elucidate its underlying pathways and clarify the clinical significance of Achilles tendon thickening.

## Graphical abstract

**Graphical Abstract:**
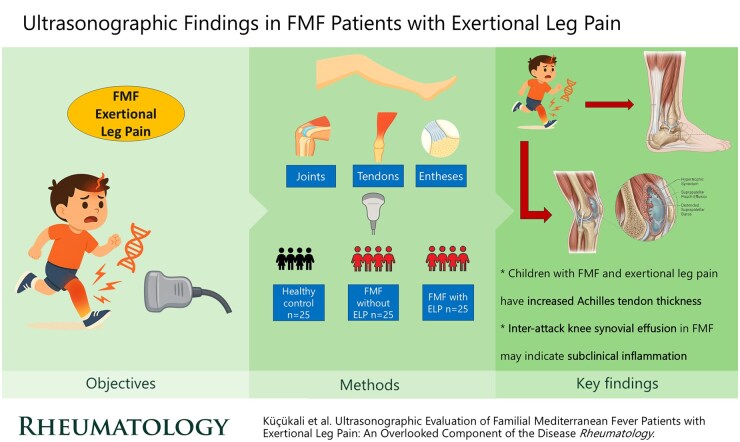

Rheumatology key messages

- Children with FMF and exertional leg pain have increased Achilles tendon thickness on ultrasonography.
- ELP reflects structural tendon adaptations that may be driven by heightened innate immunity and mechanical loading.
- Inter-attack knee synovial effusion in FMF may indicate subclinical inflammation.

## Introduction

FMF is the most common hereditary autoinflammatory disease, characterized by recurrent episodes of fever and serositis [1]. Although typical attacks include fever, abdominal pain, chest pain and joint pain, uncommon manifestations including, erysipelas-like erythema, acute scrotum, protracted febrile myalgia and exertional leg pain (ELP), may also occur [1, 2].

ELP, also referred to as exertional myalgia and exercise-induced myalgia, is a well-recognized clinical feature in children and adolescents with FMF [1, 3, 4]. It typically causes pain throughout the lower extremities following relatively mild physical activity. The reported frequency of ELP among FMF patients varies widely, ranging from 30% to 58%, primarily due to the lack of a standardized definition and its frequent under recognition in clinical practice [3–6]. Although ELP has been incorporated into international disease severity score (ISSF), it remains disregarded in current management guidelines [7, 8].

The pathophysiology of ELP is still not fully elucidated [9]. Nevertheless, it has been consistently associated with a more severe disease course [6]. Eshed *et al.* demonstrated that a subset of adult FMF patients with ELP exhibited enthesitis on MRI; however, to date, no study has radiologically investigated children with FMF and ELP [3, 10]. Herein, we investigated the joints, entheses and tendon thickness of lower extremities in children with ELP using ultrasonography and compared the findings with those of FMF patients without ELP symptoms to explore ultrasonographic correlates of ELP in paediatric FMF.

## Methods

### Target population

Patients who presented to our Pediatric Rheumatology clinic for regular follow-ups between June 2024 and March 2025, had a confirmed diagnosis of FMF according to the Eurofever/PRINTO criteria, and carried a homozygous or compound heterozygous pathogenic MEFV variant were enrolled in this prospective observational study [11]. Patients who followed for less than 1 year were not included. Those presented during FMF attack with a history of recent major lower limb trauma or with musculoskeletal complaints other than ELP were excluded. In addition, patients with any diagnosed musculoskeletal disease or those engaged in active professional sports were excluded.

ELP was defined as pain in the lower extremities occurring after relatively minor effort and resolving with rest [3, 6]. Patients were divided into two groups based on the presence or absence of ELP, referred to as ELP cohort and non-ELP cohort, respectively.

In addition, a healthy control group was included and one-to-one matched with the ELP cohort for age (by chronological year) and sex. To minimize potential confounding related to body composition, participants were balanced for BMI SDS using the national reference data and categorized according to WHO criteria [12].

### Data collection

Demographic and clinical variables, MEFV mutation status, FMF attack characteristics, treatment details, complications, haematologic parameters and acute phase reactants (CRP and serum amyloid A) were recorded. Patients’ anthropometric measurements, including weight and height, were performed in the morning, and S.D. scores were calculated according to Centers for Disease Control and Prevention (CDC) data.

Subclinical inflammation was defined as the presence of elevated acute phase reactants between attacks according to EULAR/PReS guidelines [7].

Musculoskeletal assessment was performed using the paediatric Gait, Arms, Legs, Spine (pGALS) screening tool, complemented by a thorough evaluation of the entheses, independently of ultrasonography. The ultrasonography operator was blinded to clinical findings to minimize potential observer bias [13].

### Ultrasonographic assessments

Ultrasonographic examinations were performed using a LOGIQ E scanner equipped with a 12 MHz linear transducer. B-mode and Power Doppler imaging were used according to standardized presets, with parameters optimized to obtain optimal image quality. All ultrasonographic examinations were conducted by the same paediatric rheumatologist, who has 3 years of experience in paediatric musculoskeletal ultrasonography and has completed standardized training and certification in paediatric musculoskeletal imaging.

This study was designed as an exploratory ultrasonographic evaluation of lower-extremity joints, tendons and entheses in children with FMF. Ultrasonographic evaluation of the hip, knee and ankle joints, including assessment and grading of synovial effusion and hypertrophy, as well as detection of tenosynovitis and enthesitis, was performed according to the Outcome Measures in Rheumatology (OMERACT) Ultrasound Group guidelines [14–19]. For knee joints, however, synovial effusion grading followed the standardized method described by Ting *et al.* [20] and suprapatellar effusion depth measurement was performed as outlined by Windschall *et al.* [21]. The standardized scanning protocol for each joint, enthesis and tendon thickness is illustrated in Supplementary Data S1. Joint evaluation and tendon thickness measurement protocols were adapted from previously published and widely used relevant paediatric musculoskeletal ultrasonography studies [14, 22–25].

Tendon thickness was measured at the mid-portion of the tendon rather than directly at the insertion site. This approach was preferred because the precise localization of the insertion point may be difficult in children due to thicker cartilage and anatomical variability, which may introduce measurement heterogeneity. Entheses were instead evaluated according to OMERACT definitions, including assessment of hypoechogenicity, relative entheseal thickening compared with the tendon body and structural bone abnormalities [18].

### Ultrasonographic technique

Scanning techniques for the hip, knee and ankle joints followed standardized paediatric protocols, as detailed in Supplementary Data S1.

All tendon thickness measurements were performed in two planes (longitudinal and transverse). The patellar tendon was measured 1 cm distal to the apex of the patella; the Achilles tendon, 2 cm proximal to its insertion on the calcaneus; and the plantar fascia, at its calcaneal insertion [22–25]. Patellar tendon measurements were obtained with the knee flexed at 90°, while Achilles tendon and plantar fascia measurements were performed with the ankle dorsiflexed and stabilized by the operator to ensure adequate tension and optimal image quality [25].

### Patient monitoring

Patients who exhibited pathological findings on ultrasonographic evaluation were further investigated for juvenile idiopathic arthritis/enthesitis-related arthritis (ERA). Additional tests included ANA, RF, ACPA, HLA-B27. Ultrasonographic follow-up examinations were conducted at 1-month intervals until the findings were resolved. The management consisted of NSAIDs for 2 weeks and treatment escalation for FMF if the patient had experienced one or more attacks per month during preceding 3 months or exhibits subclinical inflammation, as per EULAR/PReS recommendations [7]. Patients who did not respond or those who were positive for HLA-B27 underwent MRI of the sacroiliac joints and peripheric joint/entheses based on clinical findings.

### Data analysis

All statistical analyses were conducted using IBM SPSS software (version 26.0; IBM Corp., Armonk, NY, USA). The Shapiro–Wilk test was used to assess the normality of data distribution. For variables that did not follow a normal distribution, results were expressed as median (25th–75th percentile), while categorical variables were presented as counts and percentages. Group comparisons were conducted using the independent-samples *t* test or Mann–Whitney *U* test for continuous variables and the χ^2^ or Fisher’s exact test for categorical variables. For comparisons involving three groups, the Kruskal–Wallis test with Dunn’s *post hoc* test and Bonferroni correction was applied.

To evaluate the independent effect of ELP within FMF patients on Achilles tendon thickness, multivariate linear regression analyses were performed adjusting for age, sex and BMI SDS. Additionally, generalized linear models with normal distribution and identity link function were used to compare tendon thickness across healthy controls, ELP cohort and non-ELP cohort, with estimated marginal means and Bonferroni-adjusted pairwise comparisons. A *P*-value <0.05 was considered statistically significant. A *post hoc* power analysis was conducted based on the observed group differences. Effect sizes were calculated using Cohen’s *d*, and statistical power was estimated for significant and non-significant outcomes (*α* = 0.05).

### Intra-observer reliability

To assess measurement reproducibility, intra-observer reliability was evaluated using a random subset of stored ultrasonographic images. The same operator re-evaluated the images regarding synovial effusion and hypertrophy and measured Achilles tendon thickness in 30 randomly selected images after a 2-week interval, blinded to the initial measurements. Intraclass correlation coefficients (ICCs) were calculated to determine the level of agreement between the two measurements. The analysis demonstrated excellent intra-observer agreement (ICC = 0.93; 95% CI: 0.88–0.96).

### Ethical considerations

Ethical approval for the study was obtained from the Ethics Committee of Gazi University, in accordance with the Declaration of Helsinki (approval number: E-77082166-604.01-1002164). Written informed consent was obtained from both the parents and the patients prior to inclusion in the study.

## Results

A total of 25 healthy controls and 50 FMF patients were included, of whom 25 (50%) exhibited ELP. ELP cohort comprised 14 girls (56%), with a median age of 13.0 [11–16.9] years, while the non-ELP cohort included 11 girls (44%) with a median age of 13.7 [12.1–16.8] years. Anthropometric characteristics were comparable across the groups (Table 1). No significant differences were observed between the groups in terms of MEFV variants, including the presence of M694V or homozygous/compound heterozygous variants. However, six patients (24%) in the non-ELP cohort were receiving anti-IL-1 therapy (one on anakinra and five on canakinumab), whereas none in the ELP cohort were on biologic treatment (*P* = 0.022). However, disease severity scoring according to Pras and ISSF did not differ between the groups (*P* = 0.614 and *P* = 0.785, respectively). Demographic and clinical characteristics of the patients are summarized in Table 2. Laboratory findings indicated subclinical inflammation in 10 patients from the ELP cohort and eight patients from the non-ELP cohort.

**Table 1:** Anthropometric measurements of the healthy control and FMF cohorts.

Anthropometric measurements	Healthy control cohort *n* = 25	Non-ELP cohort *n* = 25	ELP cohort *n* = 25	*P*-value
Female, *n* (%)	14 (56)	11 (44)	14 (56)	0.618
Age, median [IQR] (years)	13.6 [11.5–16.7]	13.7 [12.1–16.8]	13 [11–16.9]	0.801
Height SDS, median [IQR]	0.56 [−1.5 to 1]	−0.24 [−1.2 to 0.38]	0.18 [−1.1 to 0.18]	0.342
Weight SDS, median [IQR]	−0.04 [−1.1 to 0.97]	−0.43 [−0.83 to 0.64]	0.17 [−0.69 to 0.8]	0.431
BMI SDS, median [IQR]	0.13 [−0.6 to 0.63]	−0.22 [−0.75 to 0.56]	0.15 [−0.55 to 0.8]	0.697

ELP: exertional leg pain; IQR: interquartile range; SDS: standard deviation score.

**Table 2:** Clinical characteristics of the patients.

Clinical characteristics	Non-ELP cohort *n* = 25	ELP cohort *n* = 25	*P*-value
Female, *n* (%)	11 (44)	14 (56)	0.396
Age, median [IQR] (years)	13.7 [12.1–16.8]	13 [11–16.9]	0.508
Age at disease onset, median [IQR] (years)	5 [3–8]	6 [4.5–8]	0.367
Duration of follow-up, median [IQR] (years)	9 [5.5–10]	8 [4–9.5]	0.230
Number of FMF attacks, last 6 months, median [IQR]	0 [0–1]	0 [0–2]	0.213
Amyloidosis, *n* (%)	1 (4)	0	1
ISSF/Pras score			
Mild, *n*	15/9	14/12	0.785/0.614
Intermediate, *n*	7/11	9/10	
Severe, *n*	3/5	2/3	
MEFV analysis			
M694V homozygous, *n* (%)	19 (76)	20 (80)	0.733
Compound heterozygous, *n* (%)	6 (24)	5 (20)	0.733
M694V mutation in at least one allele, *n* (%)	23 (92)	24 (96)	1
Laboratory findings			
Anaemia, *n*	1 (4)	0	1
Subclinical inflammation, *n* (%)	8 (32)	10 (40)	0.556
Treatment			
Colchicine, *n* (%)	25 (100)	25 (100)	1
Colchicine dose, median [IQR] (mg)	1.5 [1.2–2]	1.5 [1–2]	0.260
Anti-IL-1 treatment, *n* (%)	**6 (24)**	**0**	**0.022**

Bold text indicates statistically significant results.

ELP: exertional leg pain; IQR: interquartile range; ISSF: International Severity Score for FMF; SDS: standard deviation score.

**Figure 1:**
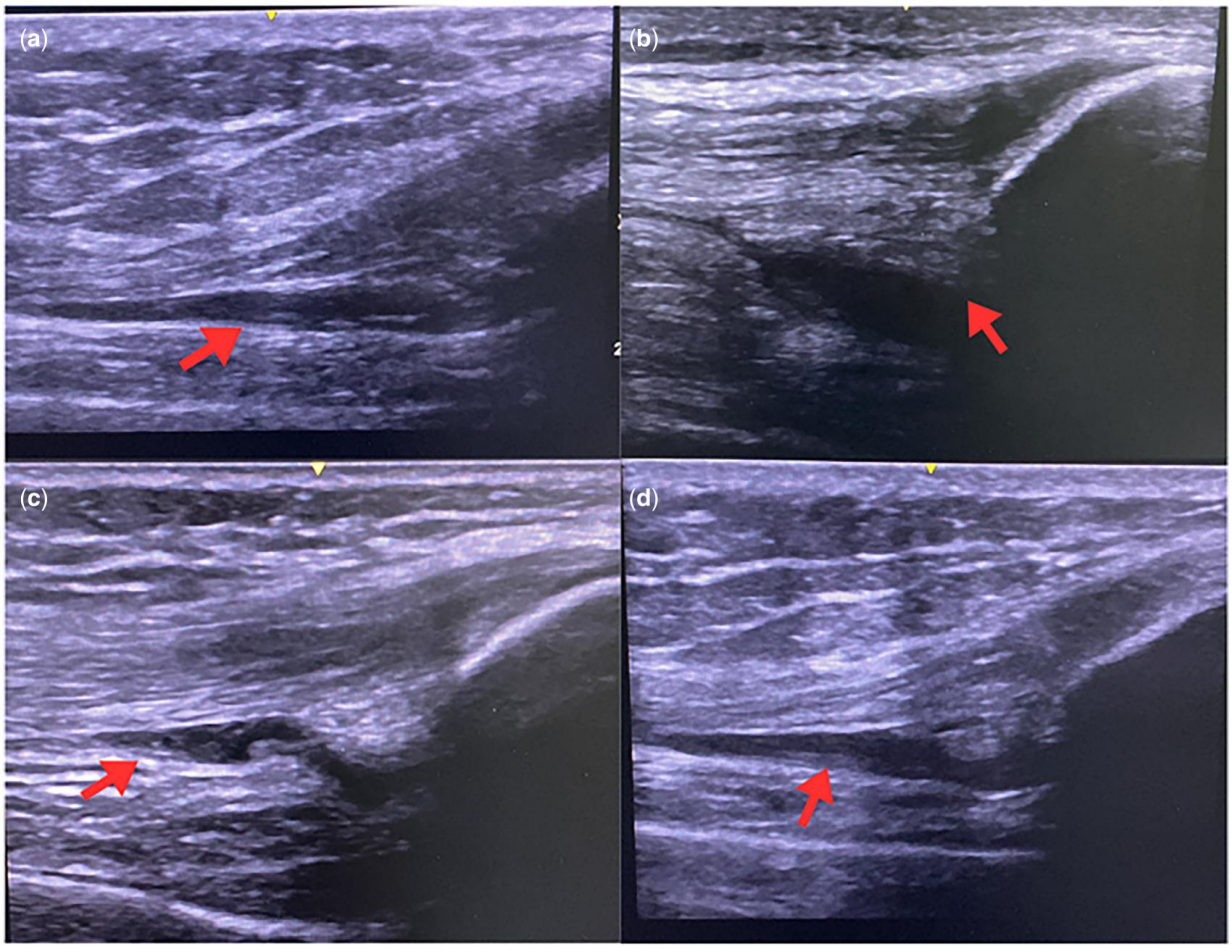
Ultrasonographic images of knee synovial effusion in patients. Images were obtained with the knee in a neutral position and the probe positioned longitudinally over the suprapatellar region. Arrows in **a** and **c** indicate synovial effusion with synovial hypertrophy, **b** highlights the displaced prepatellar fat pad, and **d** demonstrates synovial hypertrophy

**Figure 2:**
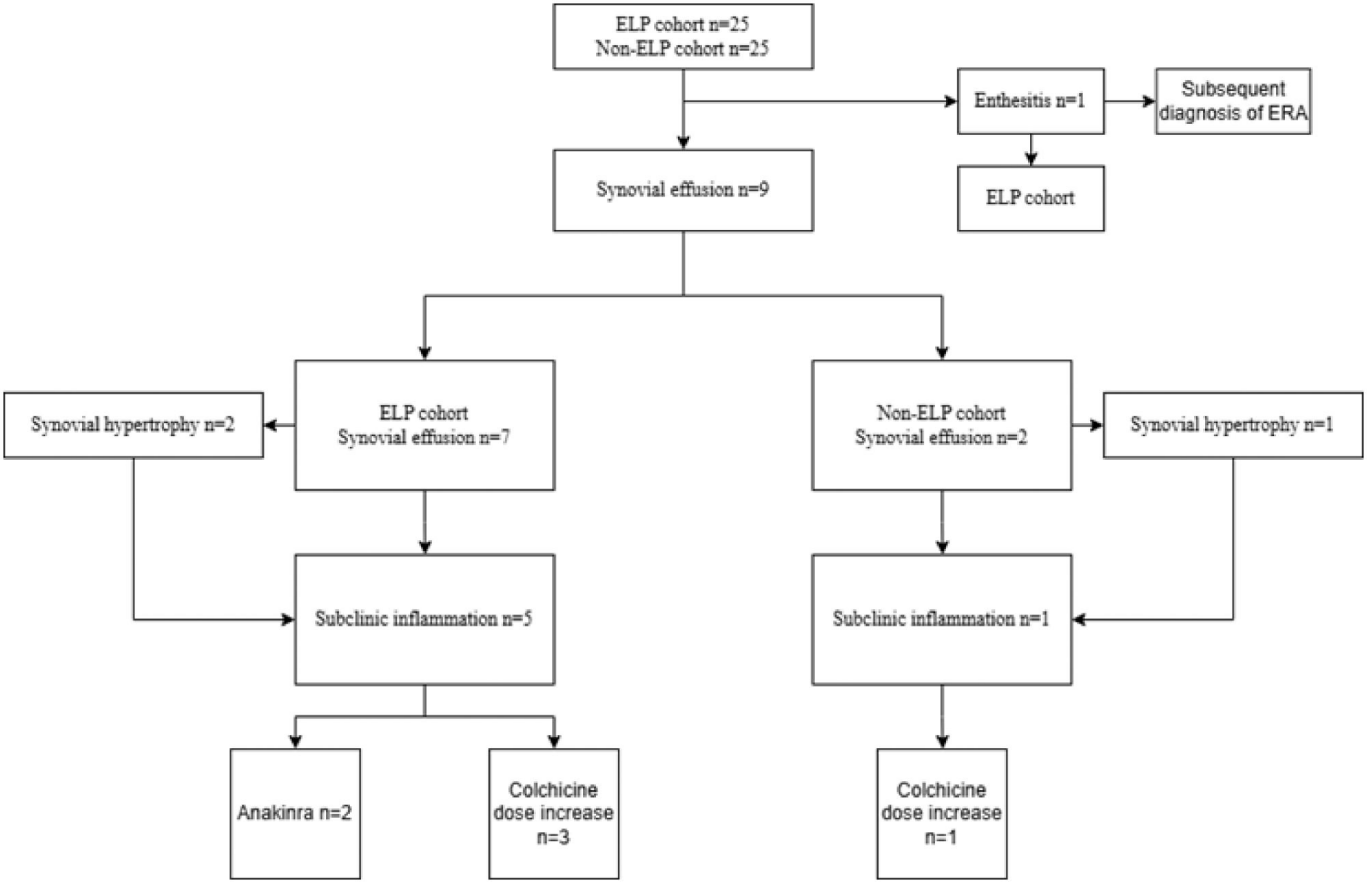
A flow diagram summarizing ultrasonographic findings, subclinical inflammation and treatment escalation. ELP: exertional leg pain, ERA: enthesitis-related arthritis

Ultrasonographic evaluation revealed knee synovial effusion in seven patients with ELP and two patients without ELP. Among these nine patients, four demonstrated bilateral knee effusions, and seven exhibited subclinical inflammation. Representative ultrasonographic images of these patients are shown in Fig. 1. All synovial effusions were classified as grade 1, and three patients from the ELP cohort with subclinical inflammation also demonstrated synovial hypertrophy [15, 20]. However, no PDI activity was detected in any patient. Of the patients with synovial effusion, seven had concurrent subclinical inflammation. Treatment escalation was therefore implemented—two patients initiated anakinra, while the others were advised to increase their colchicine dosage. At 1-month ultrasonographic follow-up, none of the patients exhibited synovial effusion after 2 weeks of NSAID therapy and FMF treatment adjustment, when indicated. Further investigations, including RF, ACPA and HLA-B27, were negative in all the cases. A flow diagram summarizing ultrasonographic findings, subclinical inflammation and treatment escalation is shown in Fig. 2.

In addition, one child in the healthy control had a minimal knee synovial effusion without synovial hypertrophy, under the pathological cut-off measurement level (2.7 mm) according to PIUS-knee study [21].

**Figure 3:**
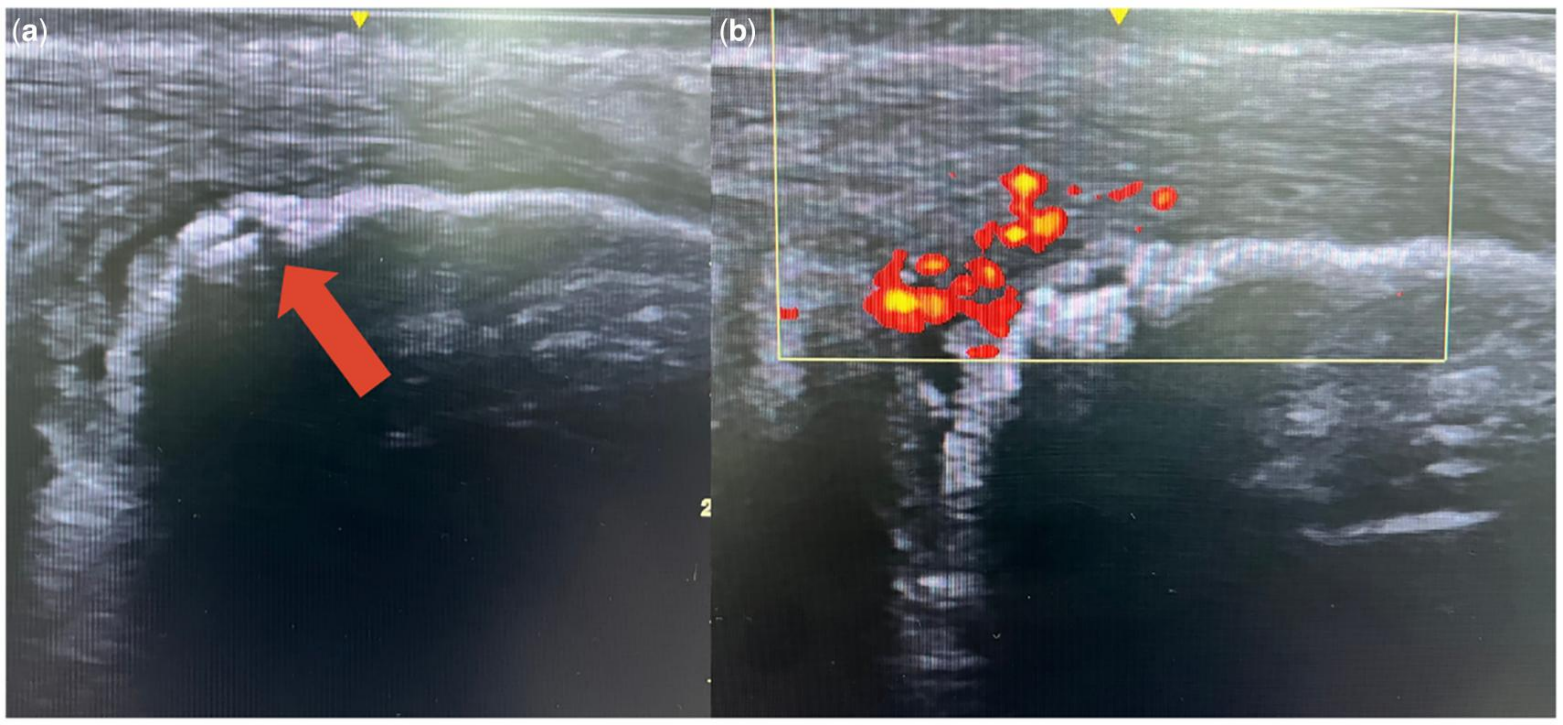
Ultrasonographic findings of the patient subsequently diagnosed as ERA. (**a**) B-mode image demonstrating bone irregularity and retrocalcaneal bursitis (arrow highlights pathological changes). (**b**) Power Doppler imaging showing increased vascular signal at the tendon and enthesis levels of the Achilles tendon

Only one patient from the ELP cohort demonstrated bone irregularity, presence of PDI both at the tendon and the enthesis levels of achilles and retrocalcaneal bursitis. Subsequent imaging, including sacroiliac and ankle MRI, confirmed sacroiliitis and findings consistent with HLA-B27-negative ERA, for which conventional disease-modifying antirheumatic therapy was initiated. The patient’s representative ultrasonographic images are present in Fig. 3.

Tendon and fascia thickness measurements revealed bilaterally increased Achilles tendon thickness in the ELP cohort compared with the non-ELP cohort and the healthy group (*P* < 0.001 for both right and left sides). *Post hoc* pairwise comparisons with Bonferroni correction confirmed that the ELP cohort had significantly thicker tendons than both the healthy controls (adjusted *P* < 0.001 for both sides) and the non-ELP cohort (adjusted *P* = 0.001 for right and *P* = 0.003 for left sides). No significant difference was observed between the non-ELP cohort and the healthy group (*P* > 0.05). However, no significant differences were observed in patellar tendon or plantar fascia thickness between the two groups. All ultrasonographic findings are summarized in Table 3.

**Table 3:** Ultrasonographic findings of the patients.

Ultrasonographic findings	Healthy control cohort *n* = 25	Non-ELP cohort *n* = 25	ELP cohort *n* = 25	*P*-value	*Post hoc* pairwise
Joint ultrasonography					
Synovial effusion, *n* (%)	**0**	**2 (8)**	**7 (28)**	**0.028**	**C vs A**
Synovial hypertrophy, *n* (%)	0	1 (4)	2 (8)	0.353	
PDI positivity, *n*	0	0	0		
Enthesitis, *n* (%)	0	0	1 (4)	0.363	
Tendon measurements					
Left patellar tendon, median [IQR], cm	0.3 [0.26–0.32]	0.29 [0.23–0.30]	0.3 [0.26–0.33]	0.365	
Right patellar tendon, median [IQR], cm	0.3 [0.27–0.33]	0.3 [0.27–0.32]	0.29 [0.26–0.34]	0.834	
Left achilles tendon, mean ± SD, cm	**0.37 ± 0.02**	**0.39 ± 0.05**	**0.44 ± 0.06**	**<0.001**	**C vs A, C vs B**
Right achilles tendon, mean ± SD, cm	**0.38 ± 0.02**	**0.40 ± 0.06**	**0.48 ± 0.08**	**<0.001**	**C vs A, C vs B**
Left plantar fascia, median [IQR], cm	0.17 [0.15–0.19]	0.17 [0.15–0.19]	0.18 [0.14–0.21]	0.752	
Right plantar fascia, median [IQR], cm	0.18 [0.16–0.19]	0.18 [0.16–0.19]	0.18 [0.16–0.21]	0.604	

Bold text indicates statistically significant results.

ELP: exertional leg pain; IQR: interquartile range; PDI: power Doppler imaging.

Generalized linear models demonstrated significant group effects on Achilles tendon thickness bilaterally (right: *χ*^2^(2) = 39.074, *P* < 0.001; left: *χ*^2^(2) = 27.287, *P* < 0.001), after adjustment for age, sex and BMI SDS. Estimated marginal means showed that the ELP cohort had significantly greater tendon thickness than both the non-ELP and healthy control groups on both sides (Bonferroni-adjusted *P* < 0.001), while no difference was observed between the non-ELP and healthy control groups. To further assess the independent contribution of ELP within the FMF cohorts to Achilles tendon thickness, multivariate linear regression analyses were performed, adjusting for age, sex and BMI SDS [26]. The model explained 40.7% and 29.8% of the variance in right and left Achilles tendon thickness, respectively, and was significant (right: *R*^2^ = 0.407, *F* = 5.483, *P* = 0.002; left: *R*^2^ = 0.298, *F* = 3.399, *P* = 0.02). ELP was identified as a significant positive predictor of tendon thickness for both sides (right: *β* = 0.576, *t* = 4.163, *P* < 0.001, 95% CI: 0.050–0.147; left: *β* = 0.525, *t* = 3.489, *P* = 0.001, 95% CI: 0.027–0.104), indicating that patients with ELP had thicker Achilles tendons, independent demographic and anthropometric factors.

In addition, exploratory analyses including IL-1 inhibitor use as an additional covariate demonstrated no significant association with Achilles tendon thickness (*B* = −0.047, *P* = 0.223). The independent effect of ELP remained significant.

## Discussion

In this prospective exploratory ultrasonographic study, we demonstrated that children with FMF who experience ELP exhibit significantly increased achilles tendon thickness compared with those without ELP and with age- and sex-matched healthy controls, suggesting that the observed tendon thickening is unlikely to represent normal anatomical variation and may instead reflect disease-related musculoskeletal involvement in FMF patients with ELP. Although tendon thickening alone is not defined as a hallmark feature of enthesitis or tenosynovitis according to OMERACT guidelines, this finding may explain the post-exertional pain and may also suggest the presence of structural tendon alterations associated with ELP [18, 19]. Furthermore, although not statistically significant, one child in the ELP cohort demonstrated subclinical enthesitis and was consequently diagnosed with ERA. Additionally, asymptomatic synovial effusion, especially when accompanied by synovial hypertrophy, may serve as a marker of subclinical disease activity and inflammation in FMF patients.

ELP has long been recognized as one of the characteristic manifestations of FMF [5]. While it was acknowledged in Tel-Hashomer diagnostic criteria and ISSF, recent diagnostic tools and management guidelines have disregarded this feature [5, 7, 11]. The absence of a standardized definition or management protocol has contributed to its under recognition among clinicians. Consequently, robust epidemiological data remain limited, but retrospective studies have reported that 30–58% of FMF patients experience ELP [3–6]. Given that ELP is often overlooked and poorly defined, its true prevalence is likely higher. A prior study investigating the pathophysiological mechanisms of ELP demonstrated increased post-exercise acidification in the gastrocnemius muscle, although these results have not been validated [9]. Moreover, ELP has been associated with a more severe disease course and with M694V homozygous with *MEFV* M694V homozygosity—both of which have also been linked to chronic musculoskeletal involvement in FMF, particularly HLA-B27-negative sacroiliitis—suggesting these may represent points along a shared pathogenic spectrum [3, 4, 27, 28]. Further MRI-based studies in adults have shown an association between ELP and enthesitis before and after treadmill exercise [3, 10]. However, no study has comprehensively investigated ELP in children beyond its correlation with disease severity [6]. Our findings did not support the previously reported association between ELP and a more severe disease course [6]. Prior studies likely reflected the higher prevalence of M694V mutations among ELP patients, as this genotype itself is linked to severe disease [3, 6]. In our cohort, both the groups predominantly carried M694V homozygosity, resulting in comparable disease severity scores despite ELP being included as a component of the ISSF.

Recent standardized paediatric joint USG protocols and interobserver validation studies have harmonized image acquisition and interpretation, successfully promoting a uniform methodology [14, 22, 29–31]. Despite the lack of internationally validated normative cut-offs, PDI-enhanced multiplanar assessment provides valuable diagnostic information with strong MRI correlation [21, 22, 32–34]. Additionally, USG can also identify arthritis in the absence of clinical findings—termed subclinical arthritis—in patients with juvenile idiopathic arthritis [35]. Therefore, USG represents a highly valuable, non-invasive diagnostic modality in paediatric rheumatology, with robust validation supporting its use in standardized assessment.

Normal reference values for Achilles tendon insertion thickness in healthy children were reported by Türkmen *et al.* [36]. However, pathological cut-offs for different age and BMI groups, particularly in ERA patients, remain undefined. Therefore, we included a healthy control group to minimize interobserver bias and to prevent measurement inconsistencies between operators, such as the inclusion of the paratenon sheath for the Achilles tendon, the aponeurosis for the plantar fascia, or variations in patient positioning during examination.

In the present study, the ELP cohort demonstrated significantly thicker Achilles tendons than both the non-ELP cohort and healthy controls. While no paediatric studies have defined clinically meaningful tendon thickness differences, adult studies have reported an average difference of ∼1.4 mm between asymptomatic individuals and those with Achilles tendinopathy [26]. Nonetheless, tendon thickness is influenced by age, sex, height and BMI; therefore, direct comparison with adult data is not appropriate [22, 26, 36]. Although increased tendon thickness is not independently defined as enthesitis by OMERACT, it may nonetheless account for pain symptoms in these patients [18].

Achilles morphology is sensitive to mechanical loading; thus, the observed right–left differences likely reflect physiological variation related to limb dominance, consistent with previous literature [36]. Interestingly, although patients with ELP might be expected to limit physical activity due to pain, the presence of increased tendon thickness suggests that additional disease-related factors may be involved rather than simple activity-dependent adaptation.

A plausible explanation for the overall bilateral tendon thickening observed in the ELP group is the interaction between repetitive mechanical stress and heightened innate immune responsiveness in FMF. FMF is characterized by dysregulated innate immune activation driven by IL-1-mediated inflammasome pathways, while the Achilles tendon is exposed to substantial mechanical loading and microtrauma during daily activities, suggesting that the ELP phenotype may represent a subgroup with increased sensitivity to routine mechanical loading [36]. In such individuals with increased innate immune sensitivity, mechanical stress may trigger a disproportionate inflammatory or reparative response, potentially leading to structural tendon adaptation and increased thickness even in the absence of overt inflammatory enthesitis. However, this interpretation remains hypothesis-generating, and our data do not directly demonstrate this mechanism.

We did not find differences in plantar fascia thickness between cohorts, which contrasts with findings in adult studies [3]. This selective increase in Achilles tendon thickness may be related to the fact that the Achilles tendon is exposed to substantial mechanical loading and repetitive microtrauma. Although preferential Achilles involvement has been described in ERA compared with adults, our data are insufficient to support any disease-specific interpretation [37]. Therefore, these findings should not be considered conclusive. In addition, the absence of significant differences in plantar fascia and patellar tendon thickness should be interpreted cautiously due to limited statistical power for small effect sizes.

Knee joint ultrasonography has been the central to the paediatric musculoskeletal ultrasonography research, as knee is the most frequently affected and most accessible joint in JIA [20, 21, 33]. Consequently, both normative reference values and characteristic pathological findings have been well established [20, 21]. It is important to note that up to 64% of healthy children may exhibit minimal physiological fluid in the suprapatellar recess, particularly during adolescents. Consistent with these reports healthy controls exhibited minimal physiological fluid. However, a minor synovial effusion, which can be categorized as Grade 1 according to Ting *et al.*, was detected in one child in our healthy control group [20]. This finding was interpreted as non-pathological based on the PIUS-knee cut-off values [21]. This observation further highlights the importance of recognizing minor anatomical variations and physiological findings, as emphasized in the literature. To avoid overinterpreting physiological fluid as pathological effusion, we adopted the 3.5 mm cut-off depth proposed by Windschall *et al.* and applied the grading system described by Ting *et al.* [20–22, 33].

In our study, nine patients had grade 1 synovial effusion in their knee joints. Although no significant difference was observed between the ELP and non-ELP groups in pairwise comparisons, synovial effusion was significantly more frequent in the ELP group compared with the healthy controls in the overall three-group analysis. Synovial effusion was more frequent in the ELP cohort, and synovial hypertrophy was observed only among ELP patients with subclinical inflammation. Notably, synovial effusion is not specific to inflammation and may also arise from mechanical factors such as trauma and overuse [21, 33]. However, none of our patients reported recent trauma, and 78% exhibited evidence of subclinical inflammation. Furthermore, all patients reported arthralgia during FMF attacks, and four—three of whom also demonstrated synovial hypertrophy—had experienced arthritis during attacks. All patients with subclinical inflammation responded favourably to escalation of FMF therapy within the first month of follow-up. Based on these observations, we hypothesize that joint effusions observed between attacks in FMF patients may reflect ongoing subclinical inflammation and may be interpreted as evidence of active disease in the absence of alternative causes such as trauma. Synovial effusion, particularly when accompanied by synovial hypertrophy, may also be linked to ELP and could represent part of the chronic musculoskeletal spectrum of FMF. Nevertheless, the clinical relevance of this finding and the potential role of joint ultrasonography as a marker of subclinical inflammation and disease activity in FMF warrant confirmation in larger, prospective studies.

This is the first study to evaluate ELP using lower-extremity ultrasonography in children. Our results differ from those of adult MRI-based studies reporting enthesopathy in 73.5% of FMF patients with ELP; in contrast, only one child (4%) in our cohort demonstrated ultrasonographic enthesitis [3, 10]. This discrepancy may result from the absence of paediatric-specific ultrasonographic definitions of enthesitis, and adaptation of adult criteria for children, which may cause low sensitivity [18]. Moreover, in our centre, any child reporting pain localized to enthesis is thoroughly evaluated, as clinicians in FMF-endemic regions are highly vigilant for ERA associated with FMF [38]. Additionally, enthesitis in children may also be subtler, and US may lack sufficient sensitivity to detect them, underscoring the need for MRI-based validation. Increased tendon thickness may represent a structural tendon alteration; however, whether this finding has any relationship with inflammatory enthesitis or future spondyloarthropathy requires further investigation. Prospective studies with long-term follow-up are required to clarify the clinical implications of this finding.

Substantial knowledge gaps remain regarding ELP and its clinical significance. Future research and consensus efforts are needed to establish a clear definition, elucidate its etiopathogenesis and guide its management. Although this study focused primarily on joint ultrasonography, enthesitis and tendon thickness, many patients report diffuse leg pain including myalgia. Thus, our findings may reflect only one aspect of the broader ELP pathophysiology. Inflammation of muscles and bones may also play a key role in this phenomenon.

### Limitations

Study limitations include the single-operator design and single-centre setting in an FMF-endemic region, which may limit the generalizability of our findings [1, 39].

Additionally, data regarding physical activity levels and limb dominance were not collected. Both factors may substantially influence tendon morphology and mechanical loading patterns of the Achilles tendon and therefore may affect absolute tendon thickness measurements and intergroup comparisons; therefore, the observed differences should be interpreted in the context of potential variability in mechanical loading patterns [22, 26]. Biological therapy use differed between the cohorts, which may represent a potential source of residual confounding. Although biological use was included in multivariate analyses and did not significantly alter the results, the possibility of unmeasured immunological effects, particularly related to long-term anti-IL-1 exposure, cannot be entirely excluded.

## Conclusion

This prospective exploratory study is the first to investigate structural correlations of ELP in paediatric FMF patients using standardized ultrasonographic assessment of lower-extremity joints, tendons and entheses. Our findings demonstrate that FMF patients with ELP exhibit significantly thicker Achilles tendons compared with both FMF patients without ELP and age- and sex-matched healthy children. These results suggest that Achilles tendon thickening is associated with ELP and may reflect disease-related structural tendon changes. Although increased tendon thickness alone is not diagnostic of enthesitis or ERA, it may represent a structural alteration potentially related to heightened innate immune responsiveness in the setting of repetitive mechanical stress in FMF patients with ELP. Moreover, one of 25 patients in the ELP cohort showed ultrasonographic evidence of enthesitis and was subsequently diagnosed with ERA.

A subset of patients—predominantly within the ELP cohort—also exhibited knee joint synovial effusion, and nearly all of these patients demonstrated subclinical inflammation. These findings suggest that inter-attack joint effusions even without any symptom or physical examination finding in FMF patients may reflect ongoing subclinical inflammation.

Future studies involving larger cohorts and longitudinal follow-up are warranted to clarify the clinical implications of ELP and its potential link to the spondyloarthropathy spectrum. In addition, molecular investigations are needed to elucidate the pathways underlying ELP and its association with tendon thickening and chronic musculoskeletal involvement in FMF.

## Supplementary Material

keag226_Supplementary_Data

## Data Availability

The data that support the findings of this study are not publicly available due to reasons of sensitivity but are available from the corresponding author on reasonable request.
