# Supplementary material for: Ultrasonographic evaluation of FMF patients with exertional leg pain: an overlooked component of the disease
Source: Rheumatology (Oxford). 2026 Apr 24;65(5):keag226. doi: 10.1093/rheumatology/keag226 (PMC13154411; doi:10.1093/rheumatology/keag226)
Supplement: keag226_Supplementary_Data [file keag226_supplementary_data.docx]

**Supplementary Data S1.** Standardized ultrasonographic techniques.

1. Knee joint protocol including suprapatellar (1a 30* and 1b neutral), parapatellar medial (1c) and lateral, and infrapatellar (1d) scan

Knee joint: The examination was conducted in two positions. Initially, with the patient supine and the knee in a neutral position, suprapatellar longitudinal and both medial and lateral parapatellar scans were performed. Subsequently, with the knee flexed at 30°, a suprapatellar longitudinal scan was obtained.


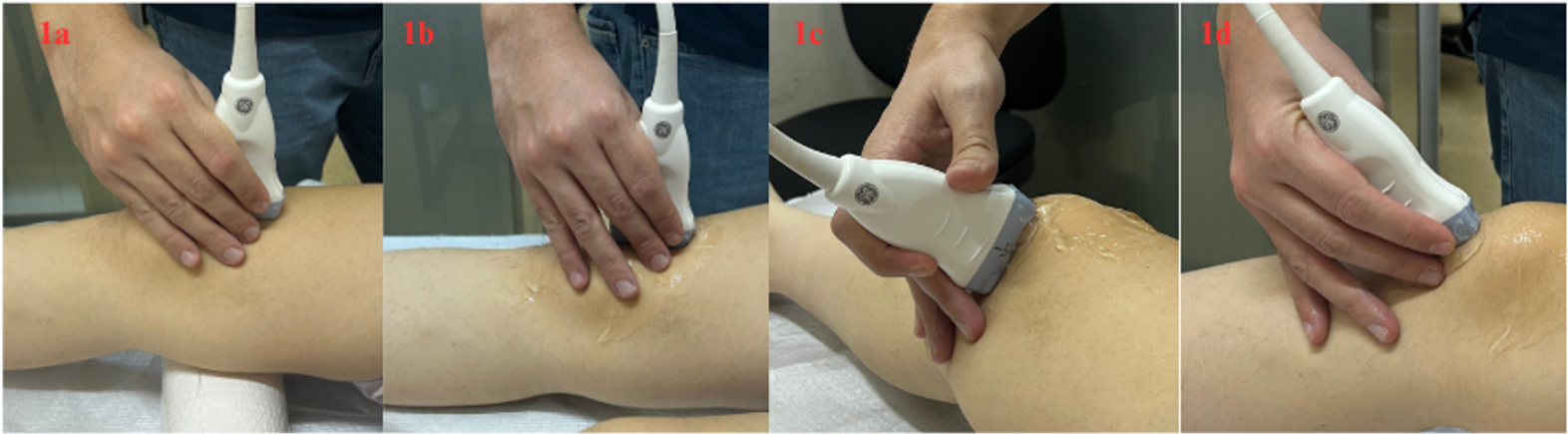


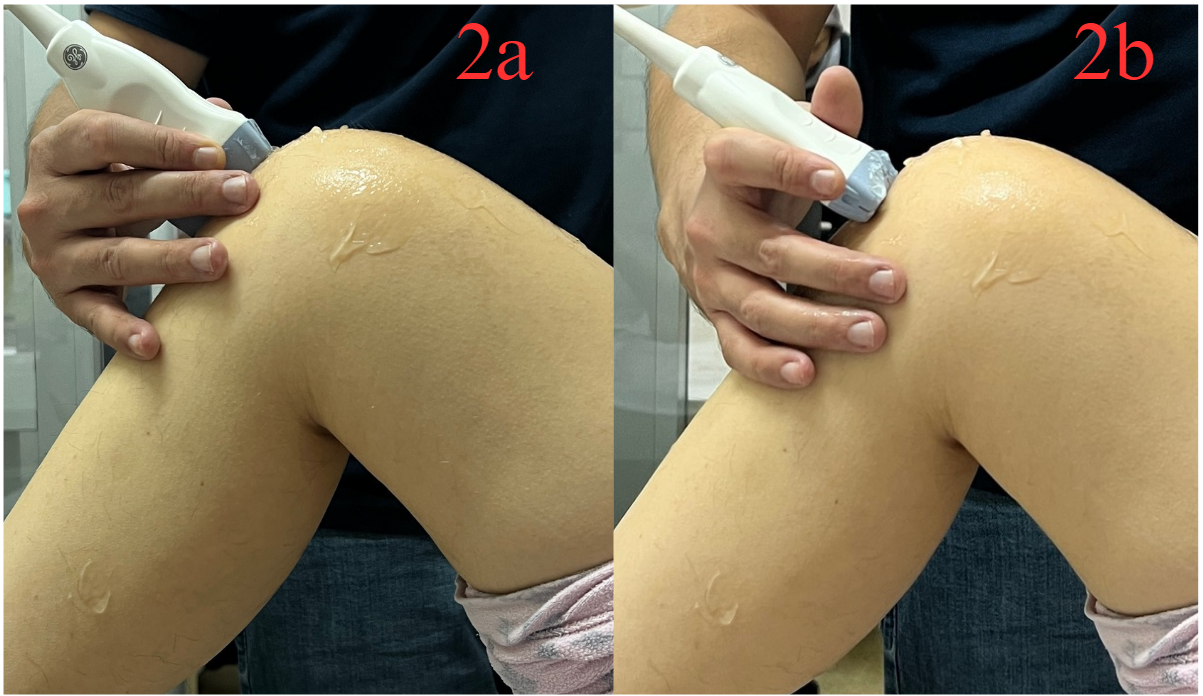
2. Two-dimensional patellar tendon thickness measurements

3. Ankle joint protocol including tibiotalar longitudinal (3a), midfoot (3b), anterior tendons (3c) subtalar medial (3c) and lateral (3d), lateral and medial ankle tendons scan.

Assessment was performed with the patient’s supine, the knee flexed at 90°, and the foot resting sole-down. A longitudinal tibiotalar scan was obtained, followed by anterior and posterior subtalar imaging during gentle eversion and inversion of the ankle.


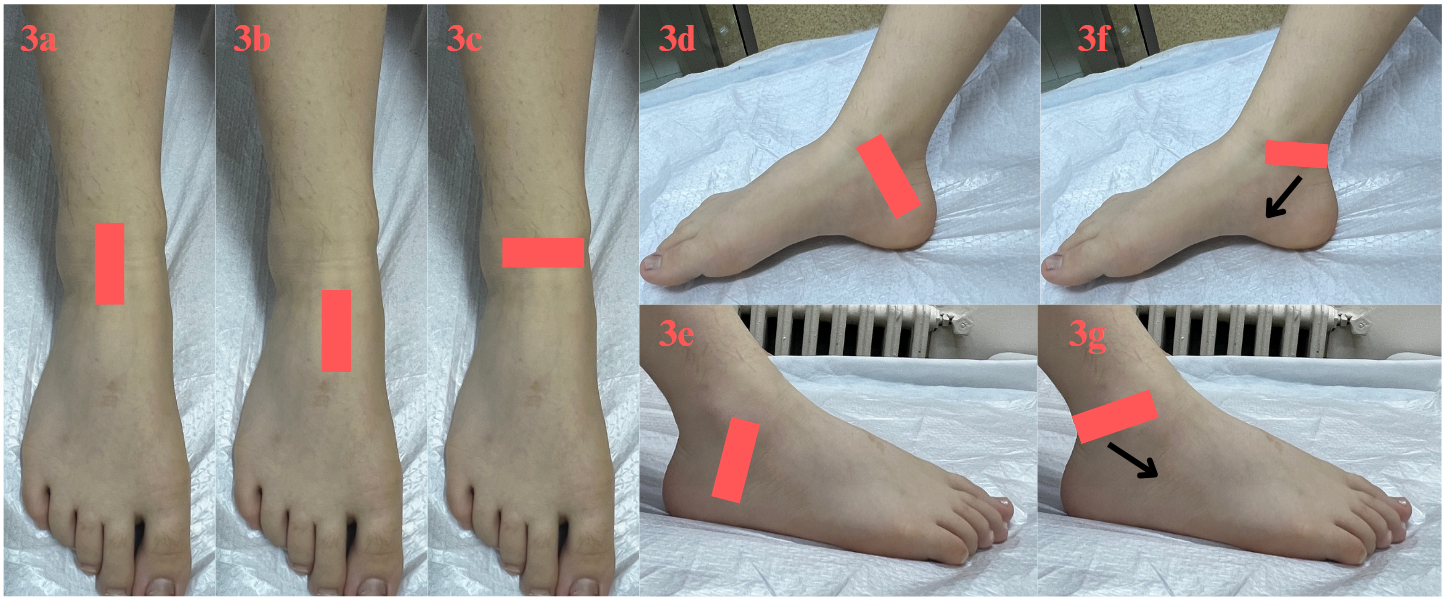


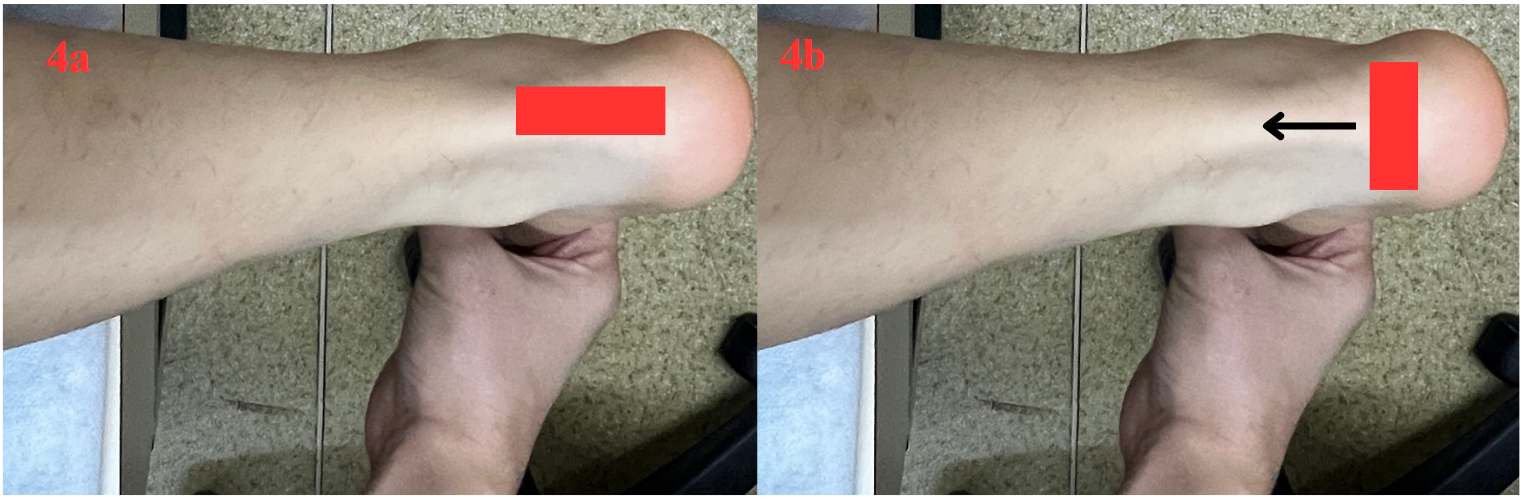
4. Two-dimensional achilles tendon thickness measurement.


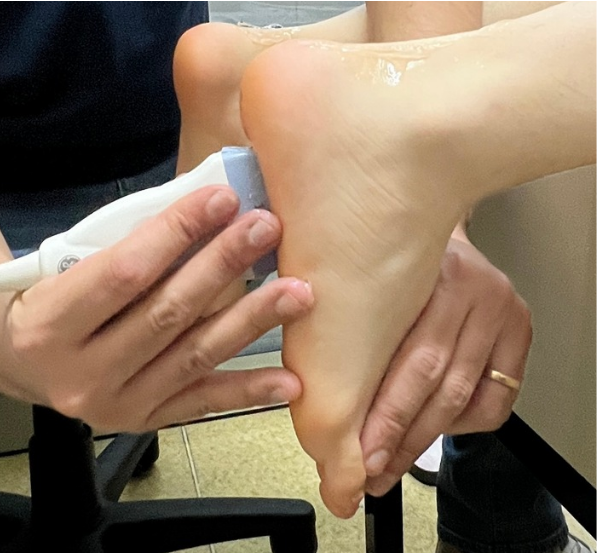
5. Plantar fascia thickness measurement.

6. Hip joint evaluation including anterior longitudinal scan.


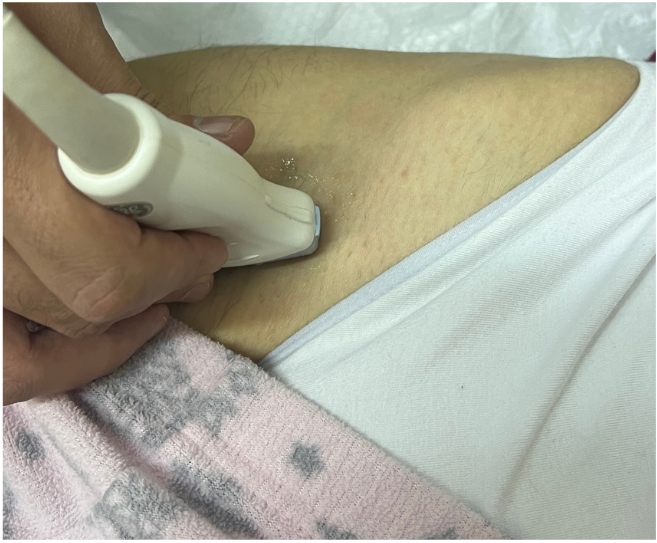
Scanning was performed with the patient in a supine position and the hip slightly externally rotated. A longitudinal anterior scan parallel to the femoral neck was obtained.
